# MRI-based radiomic feature analysis of end-stage liver disease for severity stratification

**DOI:** 10.1007/s11548-020-02295-9

**Published:** 2021-03-01

**Authors:** Jennifer Nitsch, Jordan Sack, Michael W. Halle, Jan H. Moltz, April Wall, Anna E. Rutherford, Ron Kikinis, Hans Meine

**Affiliations:** 1 Fraunhofer MEVIS Institute for Digital Medicine, Bremen, Germany; 2 Medical Image Computing Group, University of Bremen, Bremen, Germany; 3 Surgical Planning Laboratory, Brigham and Women’s Hospital, Harvard Medical School, Boston, MA USA; 4 Division of Gastroenterology, Hepatology, and Endoscopy, Brigham and Women’s Hospital, Harvard Medical School, Boston, MA USA; 5 Department of Medicine, Brigham and Women’s Hospital, Boston, MA USA

**Keywords:** End-stage liver disease, Radiomics, Biomarker, Clinical decision support

## Abstract

****Purpose**:**

We aimed to develop a predictive model of disease severity for cirrhosis using MRI-derived radiomic features of the liver and spleen and compared it to the existing disease severity metrics of MELD score and clinical decompensation. The MELD score is compiled solely by blood parameters, and so far, it was not investigated if extracted image-based features have the potential to reflect severity to potentially complement the calculated score.

****Methods**:**

This was a retrospective study of eligible patients with cirrhosis ($n=90$) who underwent a contrast-enhanced MR screening protocol for hepatocellular carcinoma (HCC) screening at a tertiary academic center from 2015 to 2018. Radiomic feature analyses were used to train four prediction models for assessing the patient’s condition at time of scan: MELD score, MELD score $\ge$ 9 (median score of the cohort), MELD score $\ge$ 15 (the inflection between the risk and benefit of transplant), and clinical decompensation. Liver and spleen segmentations were used for feature extraction, followed by cross-validated random forest classification.

****Results**:**

Radiomic features of the liver and spleen were most predictive of clinical decompensation (AUC 0.84), which the MELD score could predict with an AUC of 0.78. Using liver or spleen features alone had slightly lower discrimination ability (AUC of 0.82 for liver and AUC of 0.78 for spleen features only), although this was not statistically significant on our cohort. When radiomic prediction models were trained to predict continuous MELD scores, there was poor correlation. When stratifying risk by splitting our cohort at the median MELD 9 or at MELD 15, our models achieved AUCs of 0.78 or 0.66, respectively.

****Conclusions**:**

We demonstrated that MRI-based radiomic features of the liver and spleen have the potential to predict the severity of liver cirrhosis, using decompensation or MELD status as imperfect surrogate measures for disease severity.

## Introduction

Nearly two million people worldwide die from complications of cirrhosis each year, making the disease the 11th most common cause of death globally [1, 2]. Cirrhosis is characterized by bridging fibrosis and regenerative nodules that disrupt the normal liver architecture. Cirrhosis is the final histologic pathway for chronic liver diseases caused by alcohol, viral hepatitis, nonalcoholic fatty liver disease, autoimmune disease, and metabolic disorders. The multiple etiologies of cirrhosis differ in their prevalence which can be summarized as follows: Hepatitis C (25–40%), alcoholism (25–35%), hepatitis B (15%), nonalcoholic fatty liver disease (10%), and autoimmune hepatitis (5%) [3]. Cirrhosis is prognostically subdivided into compensated and decompensated cirrhosis, with the latter characterized by higher mortality and defined as the occurrence of at least one episode of variceal bleeding, ascites, or hepatic encephalopathy. The only current curative treatment for advanced cirrhosis is liver transplantation, which is limited by the small pool of available donor organs.

The model for end-stage liver disease (MELD) scoring system aims to stratify potential liver transplant recipients by estimating 90-day mortality. The MELD was developed by the organ procurement and transplantation network (OPTN) and the united network for organ sharing (UNOS) and was implemented in 2002 for prioritizing organ allocation. The score is a formula based on the patient’s serum creatinine (Cr), total serum bilirubin (TBIL), and international normalized ratio of prothrombin time (INR):

1 $$\begin{aligned} \text {MELD}_{(i)}= & {} 9.57 \cdot \ln \left( \text {Cr} \; \hbox {mg/dl})\right. \nonumber \\&\left. + \,3.78\cdot \ln \left( \text {TBIL} \; \hbox {mg/dl}\right) \right) \nonumber \\&+ 11.2 \cdot \ln (\text {INR})+6.43. \end{aligned}$$

The formulation of MELD was modified by UNOS in January 2016 to include serum sodium (Na) [4, 5]:

2 $$\begin{aligned} \text {MELD Score}= & {} \text {MELD}_{(i)} + 1.32 \cdot (137-\text {Na}) \nonumber \\&- \,0.033 \cdot \text {MELD}_{(i)} \cdot (137 - \text {Na}). \end{aligned}$$

MELD scores are rounded to the nearest integer and range from 6 to 40, with 6 being the lowest disease severity and 40 the highest disease severity. UNOS organ allocation also provides mechanisms to expedite transplant for conditions such as HCC in the form of exception points that are added to a patient’s MELD score. These exception points are handled on a case-by-case basis; for this reason, we do not take possible MELD exception points into account. A MELD score of 15 has been shown to be the inflection between the relative risk and benefit of transplant [6, 7].

Cirrhosis leads to successive morphological and textural tissue changes to the liver and surrounding vessels and organs. Evident characteristics of cirrhosis can include: liver surface nodularity, heterogeneous enhancement of the liver, varices, ascites, expanded gallbladder fossa, splenomegaly, and sarcopenia [3, 8]. Apart from these directly visible features, radiomic feature analysis—*radiomics*—has recently shown promising results in exploiting latent information in medical images. Radiomics has been used to identify biomarkers through quantitative image-based feature extraction and analysis. Applications include correlating derived features with patient outcomes, such as survival and response to chemotherapy and radiation [9, 10]. Furthermore, radiomic parameters related to characteristic texture and morphological heterogeneity have shown the potential to yield excellent, noninvasive prognostic factors for patient outcome. Examples include tumor phenotype analysis for risk stratification of prostate cancer as well as lung lesion characterization and predicting treatment response [11–14].

MRI-based radiomic feature extraction also comes with additional challenges due to lack of signal normalization, MRI sequence standardization, and more common acquisition artifacts [15, 16]. This makes feature repeatability (also termed stability or reproducibility in this context) between different scanner types, models, or even different software versions on the same model a research field on its own. As a consequence, MRI-based radiomic features can identify different MRI manufacturer models that are using the exact same acquisition protocol, which would confound a study such as ours focusing on disease.

For these reasons, we decided to focus our initial study exclusively on MRI data from a single center, using the same manufacturer model, scanner software version, magnetic field strength, and the exact same MRI acquisition protocol. These restrictions allowed us to maximize control while assessing the value of radiomic features.

Related research has used contrast-enhanced T1-weighted MR images to automatically assess the stage of liver fibrosis. Yasaka et al. [17] trained a deep convolutional neural network to learn characteristic image-based liver fibrosis features from contrast-enhanced T1-weighted MR images from 534 patient data sets, classifying fibrosis into the stages F0, F1, F2, F3, and F4, where a stage of F4 represents liver cirrhosis. This is similar to the research by Choi et al. where liver fibrosis staging was performed on contrast-enhanced CT images [18]. Park et al. had shown this based on liver features only (with spleen-based intensity normalization), with a model for fibrosis stage estimation [19]. Recently, He et al. demonstrated the value of radiomic features for predicting liver stiffness in children and young adults based on T2-weighted MRI without contrast agent [20]. Other researchers have attempted to improve the prognostic value of the MELD scoring system by evaluating a broader set of laboratory parameters, such as the MELD-Plus score, but do not include image-derived metrics [21].

This retrospective study aimed to determine if radiomic features derived from the MRI scans of a cirrhotic patient cohort can predict the patients’ disease severity as approximated by MELD score and presence of decompensation. Furthermore, by focusing on severity assessment of end-stage liver disease we try to predict whether a patient has already decompensated by applying the same extracted radiomic features. Compared to previous work focused on fibrosis staging, we make no *a priori* assumptions about specific manifestations of disease in imaging beyond generally detecting them in the liver and spleen. Rather, we rely on objective image-derived radiomic features with established surrogates for liver disease severity. For our radiomic feature analysis, we focused our feature extraction on liver and spleen-derived features from T1-weighted MR images. To the best of our knowledge, we are the first group to investigate an image-based biomarker for severity assessment for liver cirrhosis. The overall goal of our research is to produce a cirrhosis biomarker or radiomic signature that can be used to improve guidance in patient assessment and treatment or to supplement MELD to improve transplant prioritization.

## Materials and methods

### Patient selection, MR imaging parameters, and clinical data

**Table 1:** Demographics for cirrhosis cohort

Cohort size	$n=90$
Age (years)	$61 \pm 12$ (mean $\pm$ standard deviation)
Sex	46 male/44 female
MELD score	$9.94 \pm 3.97$ (mean $\pm$ standard deviation)
	Median 8

This was a retrospective study using MRI scans of patients with cirrhosis who were undergoing hepatocellular carcinoma (HCC) screening at Brigham and Women’s Hospital (BWH) from June 1, 2015, to June 1, 2018. Institutional Review Board (IRB) approval was obtained from Partners HealthCare. Eligible patients were screened using the Partners HealthCare Research Patient Data Registry (RPDR), which gathers clinical data from within the Partners HealthCare system [22].

This query identified 417 patients with ICD10 codes of cirrhosis, and within this cohort, we searched for patients that were scanned using a multi-parametric, fat-suppressed T1-weighted MRI scanning series on a 3 Tesla scanner (a standard protocol used for HCC screening) including a five-minute scan post-contrast injection (Gadovist^®^, Bayer HealthCare AG, Medical Care, NJ, USA; in Europe also known as Gadavist^®^). The five-minute post-contrast scan is used for radiomic feature extraction, as it represents a contrast uptake phase where cirrhotic regions within the liver are enhanced.

In total, 191 MRI scans were acquired with the above standardized protocol. Chart review for each scan was performed by two hepatologists with a combined experience of 15 years to confirm the diagnosis of cirrhosis (using clinical history, liver biopsy, elastography [23]) and to classify the presence of any liver-related decompensation (as mentioned above: defined as the presence of any ascites, variceal hemorrhage, or hepatic encephalopathy). Scans were excluded (in this cascaded order, for which the respective *n* are given) if cirrhosis could not be confirmed ($n=10$), if scans were not done on a Siemens Verio MRI Scanner (Siemens Magnetom Verio, Siemens Medical Solutions, PA, USA) ($n=31$), if parameters were missing for MELD score calculation ($n=9$), prior hepatic ablation ($n=14$), prior hepatic resection ($n=1$), prior splenectomy ($n=1$), and if patients had hepatocellular carcinoma or liver lesions larger than 10 mm ($n=0$).

**Fig. 1:**
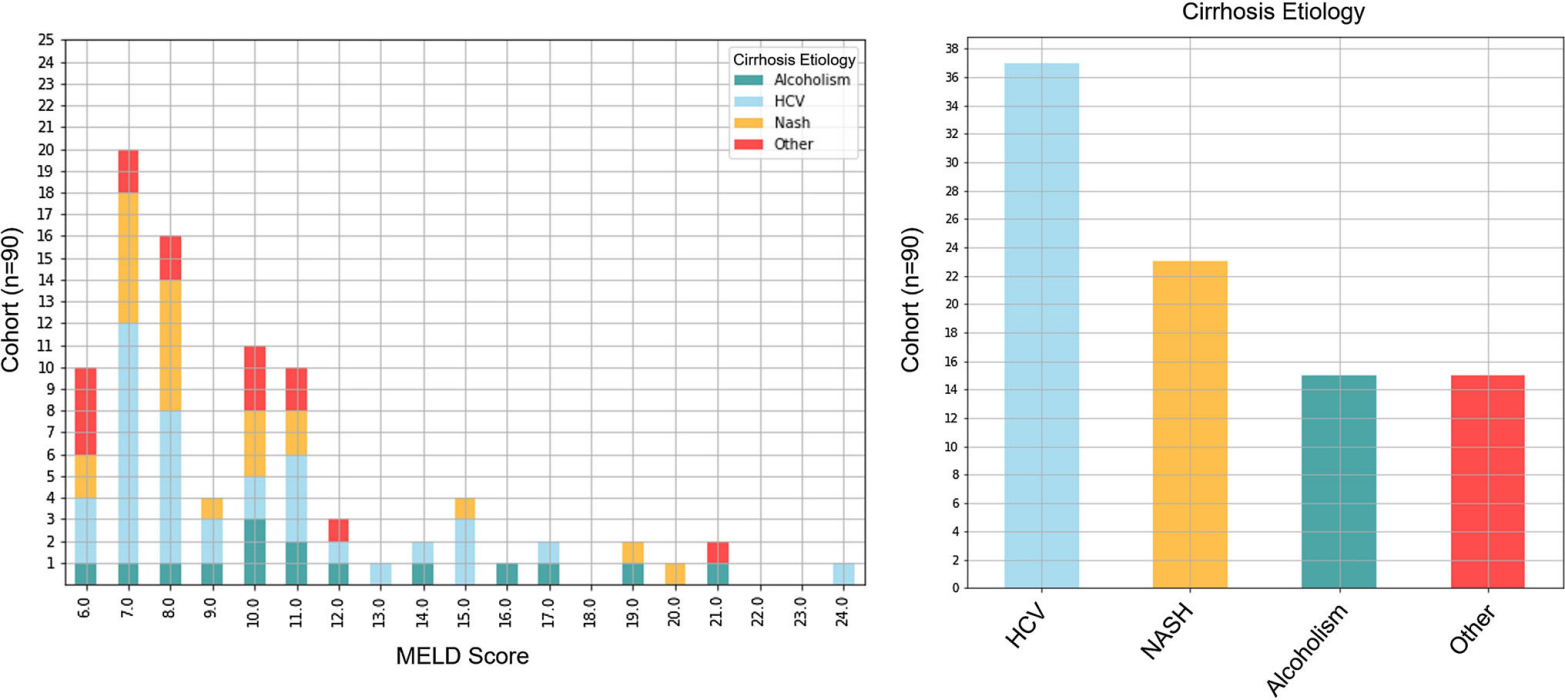
Distribution of MELD scores in liver cirrhosis cohort ($n=90$) (left) as well as an overview of different cirrhosis etiologies (right)

The final cohort consisted of 90 different patients with 125 MRI scans. If a patient had multiple scans, only the latest one scan was used for feature analysis in order to prevent a bias. The final set of images were acquired with a GRE sequence with a typical echo time of 1.79 ms, repetition time 3.79 ms, and flip angle $9^\circ$ (Siemens 3D VIBE). Contrast agent volumes were 1 ml per 10 kg body weight, up to a limit of 10 ml. All included patients obtained their MELD Labs on average within a period of ± 22 days from their MRI scan. In Table 1, we summarized the cohort’s demographic information. In Fig. 1, we give an overview of cirrhosis etiologies in our patient cohort.

### Image analysis

For quantitative radiomic feature extraction, we automatically segmented livers and spleens in our cohort using a U-net-like [24] architecture similar to Chlebus et al. [25]. The original image resolution of the fat-suppressed T1-weighted MR images acquired five minutes after contrast injection is 0.59 $\pm 0.05$ mm ranging from 0.5 to 0.86 mm with 3 mm in *z*-dimension. All images were resampled to 0.5 mm in *x*- and *y*-dimension. As preprocessing before segmentation a non-uniformity intensity correction was applied followed by a normalization to the interval [0; 1] . We started with 20 expert segmentations for training two individual neural networks for liver and spleen segmentation. Erroneous liver and spleen masks were successively corrected and the network was retrained. An expert with more than 10 years of experience in abdominal radiology validated and corrected segmented contours as necessary. Feature extraction was performed using the PyRadiomics library (version 2.0.1) in Python. For our experiments, we initially extracted features from liver and spleen segmentations using all available feature classes in the respective version for further analysis: first-order statistic features, shape-based 3D features, gray level co-occurrence matrix (GLCM) features, gray level size zone matrix features (GLSZM), gray level run length matrix (GLRLM) features, neighboring gray tone difference matrix (NGTDM) features, and gray level dependence matrix features (GLDM). Furthermore, we used LoG filters with sigma 1–5 mm. We also added the *liver-to-spleen volume ratio* as additional feature. In total, 2577 radiomic features were extracted, 1288 each for liver and spleen.

**Fig. 2:**
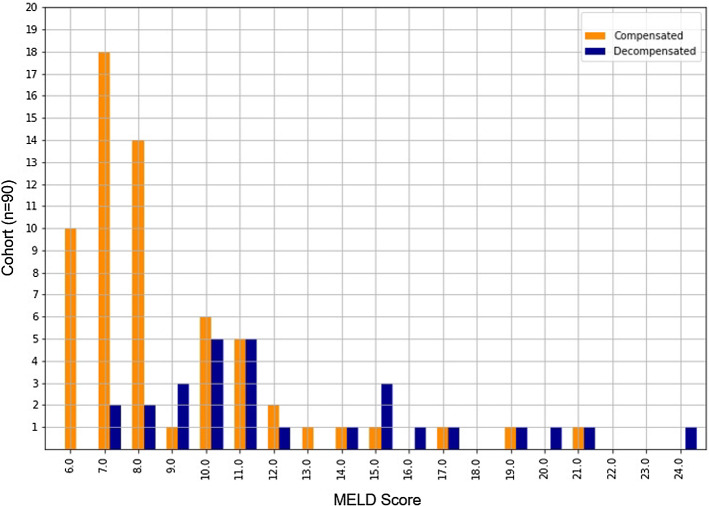
Distribution of MELD scores with an overview of compensated ($n=62$) and decompensated ($n=28$) patients

### Disease severity surrogates

We performed four different experiments in which we trained predictive models for different surrogates of disease severity:

- Experiment 1 seeks to determine a direct, MELD-score-specific prediction model of MRI-derived features with our data set (meaning: radiomic features specific for every MELD score).
- Experiment 2 attempts to create a model from MRI-derived features to predict whether a patient has a MELD score above or below the cohort median. The cohort median MELD score is 8, resulting in an almost even split at MELD score $\ge 9$ (46 patients with a lower MELD score and 44 with a higher MELD score). A MELD score of 9 has a clinical relevance as well, since a score of 10 has been suggested as a threshold at which transfer of care to a hepatologist should be considered [6, 26].
- Experiment 3 is similar to experiment 2, but attempts to predict whether a patient’s MELD score is 15 or above (where 15 represents a value where the mortality risk of transplant and cirrhosis are approximately equal). Since our patient cohort includes more patients with less advanced cirrhosis, the population of the two classes is 77 patients with a MELD $$ < 15$$ and 13 patients with a MELD $$\ge 15$$.
- Experiment 4 uses the same radiomic feature analysis, but instead uses liver decompensation (as determined by chart review) rather than MELD score as a surrogate for disease severity. Decompensation events (presence of ascites, variceal bleeding, or hepatic encephalopathy) pose severe mortality risks and impact to patient quality of life. Decompensation can be directly assessed by review of the patient’s clinical record, even in the absence of laboratory tests. The patient cohort consists of 62 compensated and 28 decompensated patients. Figure 2 shows the number of compensated and decompensated patients in the cohort for each MELD score value.

### Machine learning analysis

We used repeated ($n=15$) stratified fivefold cross-validation in each of our four experiments. For regression and classification, we employed random forests (with 100 decision trees), which have shown to be a powerful tool for machine learning analysis of radiomic features in related work [27].

We measured the performance of regression models using the coefficient of determination ($R^2$). For the classification models, we computed receiver operating characteristic (ROC) curves and measured classifier performance by means of the area under the curve (AUC).

Statistical significance of our reported AUC values was determined through a random permutation test (100 iterations, with $p <0.01$ as significance level). The classification results were aggregated from the individual cross-validation folds in which the samples were part of the test data. This enabled us to compute statistical significance for the *difference* in classification performance between separate radiomic analysis experiments, using a Wilcoxon signed-rank test on the predicted probabilities of the respective true classes.

**Fig. 3:**
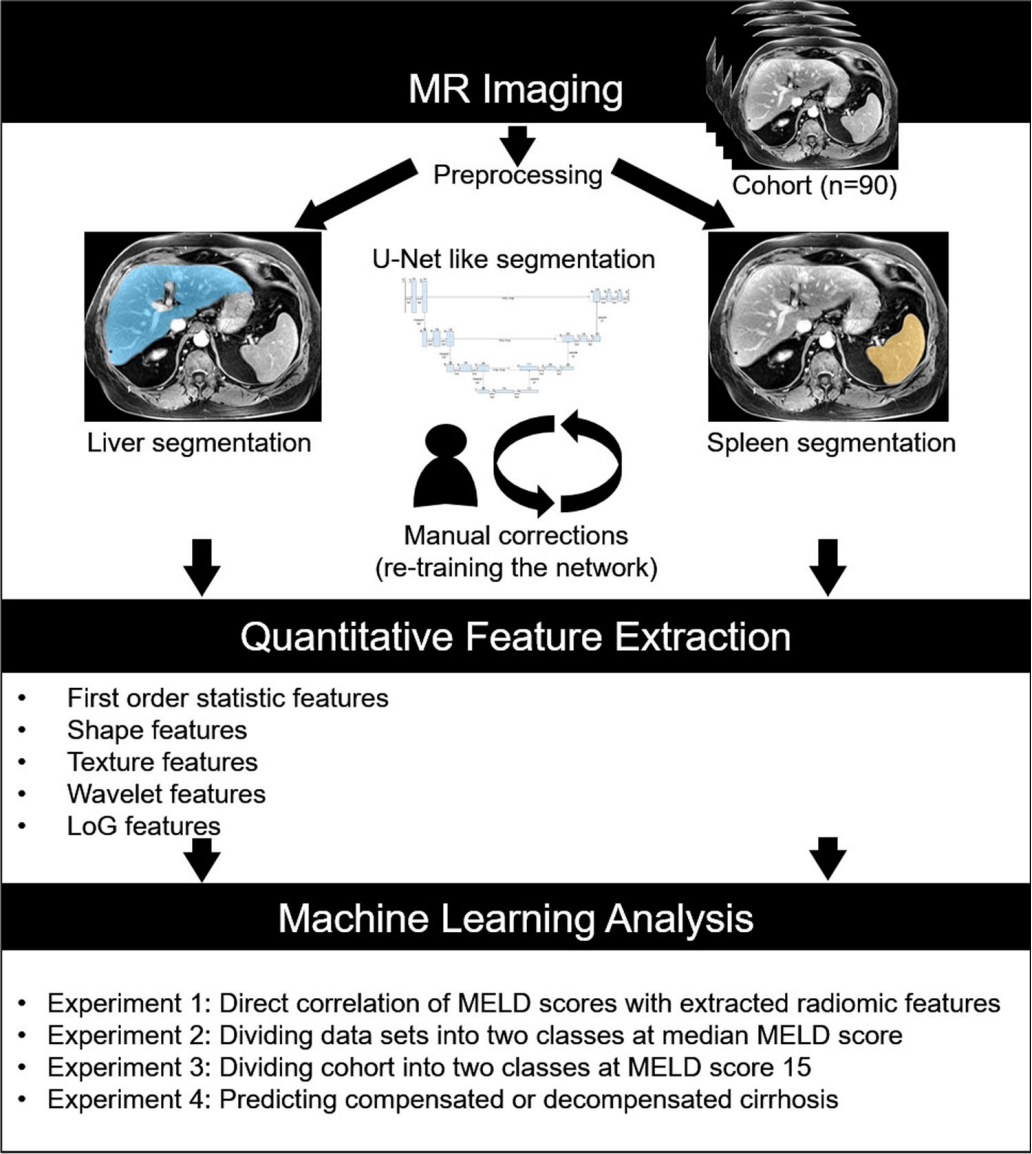
General overview of the feature extraction process and performed experiments

Figure 3 gives an overview of the general feature extraction and classification approach.

## Results

**Fig. 4:**
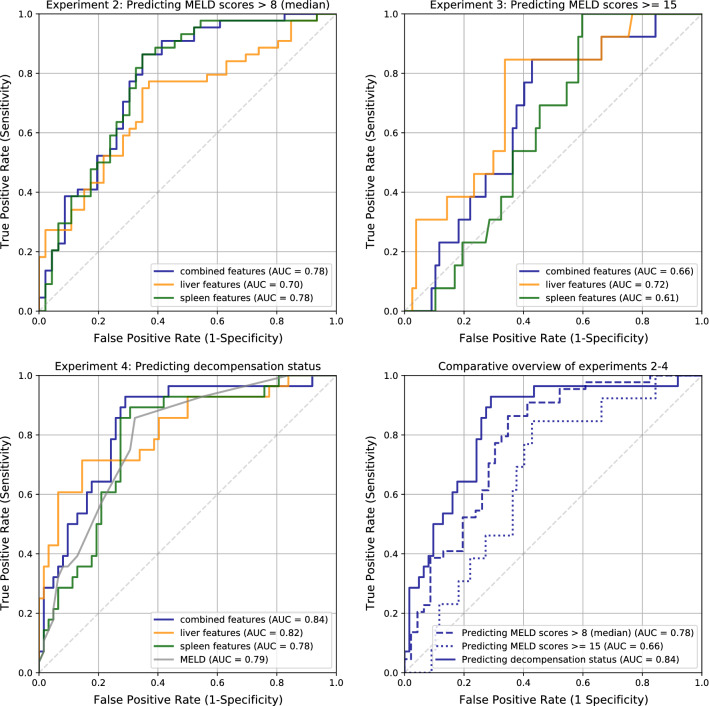
Overview of ROC curves of experiment 2–4, also comparing using liver and spleen radiomic features against solely using liver or spleen features for the classification task

In the following section, we describe the results of our experiments using radiomic feature analysis to predict different measures of cirrhosis severity. In Table 2, all results are summarized together with the ROC curves of the experiments in Fig. 4.

### Experiment 1: direct prediction of each MELD score with extracted radiomic features

For this experiment, we tested different experimental settings and approaches. As the MELD score represents integer values within the interval [6; 40] , we employed the random forest regressor. We carried out different experiments by trying to detect a correlation with just liver-derived radiomic features, spleen-derived features and trying to correlate with the ensemble of both organ features. But even after reducing the feature space by applying the FCBF feature selection method on the training data and selecting the most important features (feature selection performed on liver features, spleen features, and liver and spleen features together), we could not verify a direct correlation of radiomic features in our remaining test data sets with specific MELD scores with an $R^{2}=-0.0044$.

### Experiment 2: dividing cohort into two classes at median MELD score

Based on the observations in the previous experiment, we modified the experiment by splitting the data into two classes at the median MELD score which was 8 in our cohort. The goal was to reduce the effect of class imbalances in our relatively small cohort ($n=90$) while splitting our cohort into two categories, a lower and a higher cirrhosis disease stage. This experiment consequently transferred a regression problem to a classification problem, and the random forest classifier was applied.

With a combination of liver and spleen features, we achieved an AUC of 0.78, which was higher than with liver features alone ($\text {AUC}=0.70$, $p=0.0019$). Using only spleen features achieved an AUC of 0.78, which was significantly better than with liver features alone ($p=0.0063$) and not significantly different to using combined features. Random permutation tests showed that these AUC were statistically significant ($p <0.01$). Table 2 gives an overview of the classification results of this and the following experiments.

### Experiment 3: dividing cohort into two classes at MELD score 15

Based on the same experimental setup as in the second experiment, we evaluated a radiomic feature correlation with a split in which the higher disease stage group was defined to have MELD scores above or equal 15. We also used the same cross-validation strategy as in the previous experiment, and an AUC of 0.66 could be attained for liver and spleen features, an AUC of 0.72 with $$p <0.01 $$ when using only liver features, and an AUC of 0.61 for using solely spleen features (see Table 2). However, due to the uneven split (only 13 patients had a MELD score $$\ge 15$$), the significance test only confirmed the AUC based on liver features to be unlikely to be attained by chance (with $$p <0.01$$). Accordingly, comparisons between the different feature sets failed to show significance in the respective tests.

**Table 2:** Classification results: used radiomic features with respective area under ROC curve (AUC) and *p* values for each classification task

Experiment	Used radiomic features	AUC	*p* value
2: Split at median MELD score			
	Combined	**0.78**	$<0.01$
	Liver	**0.70**	$<0.01$
	Spleen	**0.78**	$<0.01$
3: Split at MELD score $\ge 15$			
	Combined	0.66	$<0.02$
	Liver	**0.72**	$<0.01$
	Spleen	0.61	0.13
4: Decompensation			
	Combined	**0.84**	$<0.01$
	Liver	**0.82**	$<0.01$
	Spleen	**0.78**	$<0.01$
	MELD score	**0.79**	$<0.01$

Bold values indicate the Significant results

### Experiment 4: predicting compensated or decompensated cirrhosis

The fourth experiment targets the status of liver decompensation as determined by a clinical hepatologist based on review of the electronic patient record (for details, see “Disease severity surrogates” section). Utilizing a combination of liver and spleen features for this classification task resulted in an AUC of 0.84, only using liver features led to an AUC of 0.82, and only using spleen features induced an AUC of 0.78. All single AUC values passed the significance test ($$p <0.01 $$), but the apparent difference in AUC between the usage of combined liver and spleen features versus using only liver ($$p=0.09$$) or spleen ($$p=0.2$$) features did not pass our significance level.

For reference, the MELD score itself has an AUC of 0.79 for predicting the status of liver decompensation on this same cohort (Fig. 4, gray curve).

### Feature importance

In order to determine the importance of selected features, we used the *fast correlation-based filter* (FCBF) [28]. This filter allows identification of features with minimal redundancy and maximized relevancy due to pairwise analysis of correlations between features. The resulting reduced set should contain those features that have the greatest prognostic power.

Unfortunately, given our present cohort, we could not determine a stable set of important radiomic features that were the most salient for a majority of the training and test splits within the cross-validation process.

However, we could make some qualitative observations, such as that the resulting feature sets made use of a combination of liver and spleen features. Furthermore, they always contained a different mixture of texture features (such as LoG and wavelet-based features from the feature classes GLCM, GLDM, GLSZM, and GLRLM). Most of the important LoG features were computed with a sigma of 3 or 4 mm. Shape features or the *liver-to-spleen ratio* were not ranked among the most important features. The size of the selected feature sets was about nine to thirteen, indicating that a small number of carefully selected features from both liver or spleen seem to be sufficient for the classification process.

## Discussion

In this exploratory study, we evaluated the potential for using radiomic features for severity assessment of patients with cirrhosis. Our hypothesis is that medical images of cirrhotic patients hold latent information on liver disease status that can be used to complement current clinical indicators such as MELD or decompensation. However, our experiments are complicated by the fact that no objective “ground truth” disease severity metric is available to model and evaluate against. Hence, we had to evaluate our predictive models against several existing clinical surrogates for disease severity: MELD score, MELD score $\ge$ 9, MELD score $\ge 15$, and decompensation status. As we ultimately seek to improve on current practice (which is largely based on the MELD score), perfect alignment with the existing metrics would be neither expected nor desired. Future clinical evaluation is required to fully assess the accuracy and utility of our method compared to (or in conjunction with) existing methods.

We evaluated the prognostic value of liver and spleen features together as well as solely using liver or spleen features in our experiments. Although we could not show a regression of MRI-derived radiomic features with each MELD score for severity assessment—which was an ambitious attempt from the start considering the heterogeneous patient cohort in cirrhosis etiology and disease stage, as well as our limited sample size (see Fig. 1)—we were able to show that MRI-derived radiomic features have the potential to be used for severity stratification.

We received the best results for predicting a lower or higher severity in experiment 2 and 4 if a combination of liver and spleen features is used (with limited statistical significance). For experiment 2 in which the median MELD score was used as threshold for a patient’s classification into a lower or higher disease severity, it must be mentioned that spleen features alone (AUC 0.78) have shown a higher predictive value than solely using liver features (AUC 0.70). We believe this small but statistically significant disparity ($p=0.0064$) represents a previously unreported discovery worthy of future studying. In current clinical evaluation of liver disease, the spleen is typically considered only in passing as an impression of splenomegaly or a rough measure of size by a radiologist. We believe that a more detailed analysis of spleen features is warranted and is worthy of further exploration in the diagnostic assessment of cirrhosis.

In experiment 3, a MELD score of 15 was used as threshold to define the two different classes for severity assessment, marking an important disease stage by considering if the respective patient should be listed for liver transplant. Unfortunately, given our small cohort of 90 patients, only 13 patients had a MELD of 15 or higher, which limited our ability to draw definitive conclusions and the reported AUC values did not pass the significance test.

Experiment 4 achieved the highest AUC in this work, with an AUC of 0.84 using combined liver and spleen features for discriminating between compensated and decompensated cirrhosis, meeting or modestly exceeding MELD’s predictive ability (AUC of 0.79). This result may imply that changes to the liver and spleen manifested in radiomic features align with a definition of severity defined by decompensation. While useful in clinical practice, decompensation is a crude binary measure of disease that does not provide detailed insight into the progression or severity of cirrhosis compared to, for instance, the MELD scoring system with a range for progressive severity assessment from 6 to 40. A larger patient cohort would allow a better understanding of how the liver and spleen change as patients approach and pass through the decompensation threshold. Furthermore, it must be mentioned that MELD score exception points are not handled in this work which might increase some patient MELD scores.

Moreover, experiments 2–4 demonstrate that we can train reliable, predictive models for each classification task. Even with unbalanced data sets, we demonstrate the significance of our cross-validated accuracy scores with random permutation tests. In accordance with this, the p values of experiments 2–4 are always $<0.01$ for either using liver or spleen features in the respective experiment (see corresponding p values for each experiment in Table 2). Stability and robustness of the trained predictive models can also be seen in the ROC curves in Fig. 4 within the distance of each curve to the 50% recall ratio. A fixed separation of a training and test set was not feasible in the relatively small cirrhotic cohort containing a very heterogeneous distribution of disease severity. However, to increase the general robustness of our experiments and to find stable and reliable radiomic feature for a radiomic signature a balanced data set would be desirable. Nevertheless, in our case, an overall larger patient cohort would be an additional prerequisite. The classifier needs an “adequate” number of data sets—dependent on the complexity of the classification task—in order to learn to distinguish properly between two or more classes.

According to a study published by UNOS and OPTN, the median MELD score at liver transplantation in the United States during 2018–2019 was 35 [29]. Given that our cohort was small and included patients with relatively low MELD scores, it is difficult to apply our findings to patients with higher MELD scores. Future studies that include a larger sample size for each MELD score across the entire MELD score spectrum are warranted to generate better radiomic characterization of liver disease severity.

Moreover, it has to be evaluated whether additional, objective surrogates for disease severity can be determined and included in future prediction models. For instance, the image feature analysis could be combined with other metrics derived from laboratory tests and the patient records (such as MELD, decompensation, and additional factors such as those used in MELD-Plus [21]). Statistical analysis could then be used to weight the different components by relevance to form a more wholistic clinical decision support system.

Beyond expanding the patient cohort, several additional steps will be required in order to produce a fair, objective, transparent, and widely useful radiomics-based signature or biomarker for cirrhosis severity. In particular, the differences in imaging produced at different hospitals using MRI scanners made by different manufacturers must be accounted for. Fortunately, the HCC screening protocol used in our cohort corresponds to a widely used standard in the field. In addition, our use of an open source library for radiomic feature extraction (PyRadiomics)[11] and consequently the open availability of our experimental setting should facilitate validation and extension of our work by the research community.

To the best of our knowledge, we are the first research group to analyze the prognostic value of radiomic features in this field of research. It is our hope that this work opens new avenues of research for applying radiomics and imaging to the challenges of understanding cirrhosis, treating liver disease patients, and allocating organs for transplant.
